# Commentary: “Why open-access publication should be nonprofit—a view from the field of theoretical language science”

**DOI:** 10.3389/fnbeh.2015.00201

**Published:** 2015-08-21

**Authors:** Martyn Rittman

**Affiliations:** MDPI AGBasel, Switzerland

**Keywords:** MDPI, gold open access, Languages

We read with interest the Opinion Article from Haspelmath (2013) concerning open access publication. While we agree with him that Gold open access—immediate, free dissemination of research after publication—offers an excellent route to publication for academic authors, we believe that his analysis is flawed in some aspects. In particular, he is not accurate in his characterization of MDPI and its motives.

MDPI is described by Halpelmath as a “Beijing-based.” This is factually incorrect: MDPI has been based in Basel, Switzerland since it started publishing in 1996, where the head office remains. Since 2008, MDPI has opened branch offices in China and now has three offices there—two in Beijing and one in Wuhan, but remains a registered Swiss company (see http://bs.powernet.ch/webservices/inet/HRG/HRG.asmx/getHRGHTML?chnr=2703014334&amt=270&toBeModified=0&validOnly=0&lang=4&sort=0). The same high editorial standards are applied across all our offices.

Haspelmath also comments that, “The business model here is to start a large number of new journals and to hope that some of them will succeed and bring profit.” Again, this is incorrect when applied to MDPI. In nearly 20 years of operation, we have not closed down a single journal, and all maintain a regular publication output. This is down to the dedicated work of editors, both MDPI staff and the 6500 academic editors based at universities and research institutions across the world. We operate journals across all disciplines. Our aim is to be financially sustainable, while operating a comprehensive portfolio of open access journals.

In reference to *Languages* (http://www.mdpi.com/journal/languages), Hapelmath states that “it does not even have an editor.” This statement is misleading, as it implies that the journal had already been launched without an editor in place. The facts are as follows: At the time the article was published (2013), Languages was not accepting submissions and was in the process of assembling the editorial board. Indeed, the journal website was available only via a link sent to potential editorial board members. Dr Haspelmath was one of the early invitees. We employ strict criteria when starting a journal, which include that it must have a qualified editorial board, including an Editor-in Chief. It has taken some time to arrive at this point, but there is now a full Editorial Board and an Editor-in-Chief in place, and we look forward to publishing interesting and high quality research in *Languages*.

In his opinion piece, Haspelmath gives the impression that it is self-evident that an open access publisher that charges article processing charges (APCs) must be a vanity press. However, he must surely know that to achieve sustainable success in academic publishing, one must build a reputation and publish citable articles via robust peer-review. MDPI journals fulfill these criteria, and have been trusted by scholars from the very best universities for nearly 20 years. We believe that there are benefits for an open access publisher to be either a not-for-profit organization or a commercial enterprise, and that both are viable. In the latter case, the need for financial sustainability drives efficiencies in the publication process, and innovations that add value for authors and readers alike. The lower average income of open access publishers compared to subscription publishers bears this out. We accept that there are a small minority of publishers that engage in questionable, unethical, or even illegal practices, but we do not believe that these companies will be able to sustain themselves on a long-term basis. We also believe that the majority of scholars are able to differentiate reputable publishers from those with questionable practices.

In conclusion, we invite Martin Haspelmath to revise his opinion of MDPI and accept that a commercial open access publisher can have motivations other than profit, and is well-capable of producing high quality research that is open and available for all.

## Conflict of interest statement

The author declares that the research was conducted in the absence of any commercial or financial relationships that could be construed as a potential conflict of interest.
